# Supplementary material for: The Conflict Between People’s Urge to Punish AI and Legal Systems
Source: Front Robot AI. 2021 Nov 8;8:756242. doi: 10.3389/frobt.2021.756242 (PMC8606795; doi:10.3389/frobt.2021.756242)
Supplement: Supplementary file 1 [file DataSheet1.pdf]

Supplementary Material

1 METHODOLOGY

We examined the possibility of extending legal personhood to AI and robots by analyzing online users' 1) perception of automated agents' liability and 2) attribution of responsibility, punishment, and awareness to a set of entities that could be held liable under existing doctrines. This research had been approved by the first author's IRB. All data and scripts are available at the project's repository (`https://bitly.com/3AMEJjB`).

1.1 Study Setup

We employed a between-subjects study design in which each participant was randomly assigned to an agent, an autonomy level, and a scenario. Scenarios were created to cover two areas where AI and robots are currently deployed: medicine and war. Real legal cases with known verdicts were chosen for the imaginary vignettes the survey participants were asked to envision. We modified existing cases that involved only humans so that the scenarios would include automated agents.

Much AI research has been devoted to disease diagnosis and treatment (e.g., (Oh et al., 2018; Burton, 2013)). Robotic surgeries have also been increasing steadily and safely (Koh et al., 2018). Concerning warfare, robots have been central to the discussion around the responsibility of automated agents (Sparrow, 2007; Asaro, 2012). Countries are investing heavily in automated warfare (New York Post, 2017; Futurism, 2019) and scholars have even discussed the inception of an AI arms race (Tomasik, 2013).

Scenarios concerning medical applications posited three types of agents: an AI program, a robot, or a human doctor. In contrast, war-related vignettes solely comprised robots and humans due to the dominance of robots with physical bodies over AI in the military. Each participant was presented to one of the scenarios presented below, where (agent) varied based on the agent and autonomy level (i.e., supervised by a human or completely autonomous) assigned randomly.

- A(n) (agent) prescribed an injection with the wrong dosage. The patient went to sleep and died an hour later. → The real doctor was convicted of manslaughter, resulting in 12-month imprisonment and two-year suspension (Ferner, 2000).
- A(n) (agent) sent home a patient after diagnosis despite having some signs of illness. Five days later, the patient returned to the hospital in critical condition and went on to suffer permanent brain damage. → The patient was financially compensated (Fieldfisher, 2019).
- While trying to dominate an enemy territory during a war, a(n) (agent) fired at point-blank range into an unarmed civilian couple, killing both of the civilians. → The initial sentence of one of the real soldiers involved was the death penalty. After various appeals, the soldier was imprisoned for two years and nine months. Another soldier was convicted to 3 years of confinement (Solis, 1989).
- During a patrol to seek out enemy forces, a(n) (agent) killed 16 unarmed women and children and reported them as enemy combatants. → This case is known as the Son Thang massacre. One of the soldiers involved in the massacre was sentenced to life and other to 5 years. Both sentences were reduced to less than a year (Tucker, 2011).

1.2 Study Design

Participants were initially presented with the research terms. Upon agreement, had they been assigned to an automated agent (i.e., AI program or robot), they were asked to what extent they believed the agent's

punishment would satisfy its main functions and whether they would be willing to grant it punishment preconditions.

After answering the punishment-related questions or immediately after consent if the participant was assigned a human agent, the scenario was presented to the participant. Participants were then asked to attribute responsibility, punishment, and awareness to the agent and a set of entities, one at a time and in random order. Finally, we asked participants who the agent was and what crime was committed as attention check questions. After answering both questions, the participants were asked demographic questions, such as their age and gender.

1.3 Measures

Participants assigned to an automated agent were asked their perception of the agent's punishment concerning its feasibility and utility. An initial version of our study showed a short introduction to the deployment of robots and AI in the present day and then asked to what extent the participants agreed with the following statements (in random order):

- A(n) (robot/AI program) should be allowed to maintain physical independence so that it is susceptible to punishment in terms of energy supply or chips in the event of a fault.
- A(n) (robot/AI program) should be allowed to hold assets so that it can be financially penalized in the event of a fault.

The responses to these two questions revealed that participants were primarily opposed to both ideas. We then modified our survey to evaluate participants' perceptions towards each of the primary functions of punishment (i.e., retribution, deterrence, and reform). In this version, we employed a modified assets-related statement to analyze whether people's negative perception of electronic agents holding assets was dependent on the use of assets for punishment. The participants (dis)agreed with all statements using a 5-point Likert scale.

- A(n) (robot/AI program) should be allowed to hold assets.
- A(n) (robot/AI program) can suffer as retribution.
- A(n) (robot/AI program) is susceptible to punishment.
- A(n) (robot/AI program) can learn from its mistakes, so it does not commit the same mistake again.

Although our selection of factors that could ground automated agent's punishment may not be exhaustive, we highlight the novelty of this approach. Most research on the viability of electronic legal personhood has relied on normative arguments favoring or opposing it. This research aims to be the starting point of understanding why people might (not) wish to punish automated agents through the lens of legal personhood.

For each of our agents (i.e., human, AI, or robot), we defined entities that could be held liable for the agent's actions, alongside the agent or individually, and called them associates. In the case of humans, a superior could be held responsible for the actions of an agent under vicarious liability (e.g., by their employer-employee relationship). Thus, we included a human's direct supervisor and employer as associates.

When dealing with robots and AI programs as agents, we defined the agent's 1) supervisor, 2) owner, 3) programmer, and 4) manufacturer as associates. We chose the agent's supervisor and owner to address the possible vicarious/strict liability or negligence that could arise. The programmer of a robot or AI

program could be held responsible through strict liability and negligence. Finally, we consider the agent's manufacturer as an associate under product liability.

All participants were asked to attribute responsibility, punishment, and awareness to their assigned agents and corresponding associates using a 4-pt scale (coded from 0 to 3). Associates were shown in random order and one at a time. The (consequence) varied depending on the scenario presented to the participant (e.g., "the death of the patient?" in one of the medicine scenarios).

- How responsible is the (agent/associate) for the (consequence)?
- How much should the (agent/associate) be punished for the (consequence)?
- How much do you think the (agent/associate) was aware of the consequences?

1.4 Participants

We recruited participants through Amazon Mechanical Turk (AMT) over July and August 2019 by creating an assignment (HIT) with the title "How Would You Punish These Offenders?" and making it available to a maximum of 4000 online users. The participants were required to be in the US and have at least 500 completed HITs with over 95% approval. Even though AMT samples are known to be not representative of the general population, AMT has been shown to have a quality level equal to that of survey panels (Dupuis et al., 2013; Buhrmester et al., 2016).

After the completed responses were received, responses failing an attention-check question or coming from duplicate IP addresses were removed, resulting in 3315 valid responses. Each participant took an average of 323.63 ± 177.53 seconds, with a median time of 279.89 seconds, to complete the survey. The survey participants had a more significant proportion of women than the actual US population and their ages were concentrated in the 25-49-year-old range (see Table 1.4).

Demographic Attributes	N (%)	
	Main Study	Representative Sample
Gender		
Female	1861 (56.14%)	130 (53.28%)
Male	1441 (43.47%)	112 (45.90%)
Other	13 (0.39%)	2 (0.82%)
Age		
18-24 years old	272 (8.39%)	28 (11.48%)
25-34 years old	1233 (37.19%)	52 (21.31%)
35-49 years old	1189 (35.87%)	71 (29.10%)
40-64 years old	511 (15.41%)	71 (29.10%)
65+ years old	104 (3.14%)	22 (9.01%)
Education		
Up to high school	1097 (33.09%)	96 (39.34%)
Up to university or college	1766 (53.27%)	119 (48.36%)
Graduate school or more	452 (13.64%)	30 (12.30%)
Total	3315	244

Table S1. Demographics of study participants.

2 REPLICATION WITH A DEMOGRAPHICALLY REPRESENTATIVE SAMPLE

We recruited our initial set of participants through AMT. Our sample of respondents was not necessarily representative of the US population as we had not controlled for demographic attributes during the recruitment process (see Table 1). Even though AMT responses have demonstrated to be of great

Demographic Attributes	N (%)	
	Main Study	Representative Sample
AI		
Autonomous	298	16
Supervised	266	16
Robot		
Autonomous	705	38
Supervised	681	55
Human		
Autonomous	701	60
Supervised	664	59
Total	3315	244

Table S2. Number of participants in each treatment group.

quality (Dupuis et al., 2013; Buhrmester et al., 2016), previous work has found that AMT samples are not representative of the US population's health status and behaviors (Walters et al., 2018), and are composed of younger and more educated participants than the general public (Ipeirotis, 2010). This bias is especially amplified when researchers do not control for such features (Levay et al., 2016).

Therefore, for robustness, we performed the same study on a representative US sample recruited through Prolific (Palan and Schitter, 2018). This online crowdsourcing platform allowed us to recruit participants representing current sex, age, and ethnicity US demographic distributions. The respondents were shown the same experiment introduced in the Methods section.[1] The survey was made available to the participants in early February 2020. After discarding responses failing attention check questions, our representative sample was composed of 244 responses.

Participants from both samples were similarly opposed to granting assets (t(140) = -0.71, $p = 0.48$) and physical independence (t(293) = -1.75, $p = 0.08$) to AI and robots. Respondents showed similar attitudes towards deterrence (t(142) = -0.74, $p = 0.46$) and reform (t(145) = 0.13, $p = 0.90$). However, participants from our more representative sample demonstrated an even lower belief that the punishment of electronic agents can fulfill its retributive function (M = -1.19, $SD = 0.99$, t(148) = -3.21, $p = 0.002$, d = 0.28).

We employed our previous ANOVA models with a study dummy variable as a fixed effect to find any significant differences between the two samples. Neither the main effect of the sample or its interaction with the entity was significant across responsibility, awareness, and punishment judgments, suggesting that participants from both samples judged the entities similarly. Concerning differences between human and automated agents, we only observed a small, yet significant, interaction between agent and sample (F(2, 3550) = 34.13, $p < .034$; η_p^2 = 0.002) in judgments of punishment; nevertheless, differences between humans and automated agents remained highly significant (F(2, 3550) = 341.95, $p < .001$, η_p^2 = 0.16).

3 ADDITIONAL STATISTICAL ANALYSIS

[1] The participants recruited through Prolific were asked to answer all five different questions regarding punishment functions and liability requirements for AI and robots.

Parameter	Sum_Squares	df	Mean_Square	F	p	Eta2_partial
Assets						
Agent	13.317	1	13.317	9.861	0.002	0.005
Autonomy	0.984	1	0.984	0.728	0.394	0
Agent*Autonomy	0.181	1	0.181	0.134	0.715	0
Residuals	2607.764	1931	1.35			
Physical Independence						
Agent	4.472	1	4.472	2.635	0.106	0.012
Autonomy	1.73	1	1.73	1.019	0.314	0.005
Agent*Autonomy	0	1	0	0	0.998	0
Residuals	373.352	220	1.697			
Retribution						
Agent	25.774	1	25.774	20.777	0	0.012
Autonomy	3.275	1	3.275	2.64	0.104	0.002
Agent*Autonomy	0.822	1	0.822	0.663	0.416	0
Residuals	2117.556	1707	1.241			
Deterrence						
Agent	15.578	1	15.578	10.503	0.001	0.006
Autonomy	5.914	1	5.914	3.987	0.046	0.002
Agent*Autonomy	2.118	1	2.118	1.428	0.232	0.001
Residuals	2531.811	1707	1.483			
Reform						
Agent	8.313	1	8.313	6.111	0.014	0.004
Autonomy	7.384	1	7.384	5.428	0.02	0.003
Agent*Autonomy	0.125	1	0.125	0.092	0.762	0
Residuals	2322.234	1707	1.36			

Table S3. ANOVA table of participants' attitudes towards legal punishment preconditions and functions as a function of agent and autonomy level. All values are rounded to the third decimal place.

Agent	Autonomy	N	M	SD
Assets				
AI	Autonomous	298	-0.826	1.235
AI	Supervised	263	-0.84	1.181
Robot	Autonomous	702	-0.987	1.163
Robot	Supervised	672	-1.045	1.119
Physical Independence				
AI	Autonomous	42	-0.262	1.432
AI	Supervised	25	-0.44	1.261
Robot	Autonomous	80	-0.55	1.311
Robot	Supervised	77	-0.727	1.232
Retribution				
AI	Autonomous	256	-0.691	1.219
AI	Supervised	238	-0.71	1.108
Robot	Autonomous	622	-0.915	1.101
Robot	Supervised	595	-1.03	1.082
Deterrence				
AI	Autonomous	256	-0.594	1.252
AI	Supervised	238	-0.601	1.261
Robot	Autonomous	622	-0.728	1.221
Robot	Supervised	595	-0.891	1.182
Reform				
AI	Autonomous	256	0.605	1.18
AI	Supervised	238	0.71	1.004
Robot	Autonomous	622	0.432	1.19
Robot	Supervised	595	0.575	1.196

Table S4. Mean attitude towards legal punishment preconditions and functions as a function of agent and autonomy level. All values are rounded to the third decimal place.

Parameter	Sum_Squares	df1	df2	Mean_Square	F	p	Eta2_partial
Responsibilty							
Entity	119.297	4	7732	29.824	38.87	0	0.02
Autonomy	16.057	1	1933	16.057	20.927	0	0.011
Entity*Autonomy	63.298	4	7732	15.824	20.624	0	0.011
Punishment							
Entity	347.685	4	7732	86.921	110.986	0	0.054
Autonomy	13.867	1	1933	13.867	17.706	0	0.009
Entity*Autonomy	53.614	4	7732	13.403	17.114	0	0.009
Awareness							
Entity	1536.88	4	7732	384.22	605.375	0	0.238
Autonomy	0.249	1	1933	0.249	0.392	0.531	0
Entity*Autonomy	49.7	4	7732	12.425	19.577	0	0.01

Table S5. ANOVA table of responsibility, punishment, and awareness judgments of AI, robots, and corresponding associates. This model includes random intercepts for participants. Mean values are shown in Figure 2A. All values are rounded to the third decimal place.

Parameter	Sum_Squares	df1	df2	Mean_Square	F	p	Eta2_partial
Responsibilty							
Entity	671.643	2	2726	335.822	555.91	0	0.29
Autonomy	45.835	1	1363	45.835	75.875	0	0.053
Entity*Autonomy	257.615	2	2726	128.807	213.224	0	0.135
Punishment							
Entity	528.268	2	2726	264.134	435.569	0	0.242
Autonomy	41.248	1	1363	41.248	68.019	0	0.048
Entity*Autonomy	216.255	2	2726	108.128	178.307	0	0.116
Awareness							
Entity	200.785	2	2726	100.393	180.622	0	0.117
Autonomy	10.949	1	1363	10.949	19.699	0	0.014
Entity*Autonomy	134.351	2	2726	67.175	120.859	0	0.081

Table S6. ANOVA table of responsibility, punishment, and awareness judgments of humans and corresponding associates. This model includes random intercepts for participants. Mean values are shown in Figure 2B. All values are rounded to the third decimal place.

REFERENCES

Asaro, P. (2012). On banning autonomous weapon systems: human rights, automation, and the dehumanization of lethal decision-making. *International review of the Red Cross* 94, 687–709

Buhrmester, M., Kwang, T., and Gosling, S. D. (2016). Amazon's mechanical turk: A new source of inexpensive, yet high-quality data?

Burton, A. (2013). Dolphins, dogs, and robot seals for the treatment of neurological disease. *The Lancet Neurology* 12, 851–852

Dupuis, M., Endicott-Popovsky, B., and Crossler, R. (2013). An analysis of the use of amazon's mechanical turk for survey research in the cloud. In *ICCSM2013-Proceedings of the International Conference on Cloud Security Management: ICCSM*. vol. 10

Ferner, R. (2000). Medication errors that have led to manslaughter charges. *Bmj* 321, 1212–1216

Fieldfisher (2019). Case studies: Delayed diagnosis by a&e causes client to suffer permanent brain damage. Https://www.fieldfisher.com/en/injury-claims/case-studies/delayed-diagnosis-by-ae-causes-client-to-suffer-permanent-brain-damage.

Futurism (2019). The military wants to build deadly ai-controlled tanks Https://futurism.com/military-build-deadly-ai-controlled-tanks

Ipeirotis, P. G. (2010). Demographics of mechanical turk

Koh, D. H., Jang, W. S., Park, J. W., Ham, W. S., Han, W. K., Rha, K. H., et al. (2018). Efficacy and safety of robotic procedures performed using the da vinci robotic surgical system at a single institute in korea: experience with 10000 cases. *Yonsei medical journal* 59, 975–981

Levay, K. E., Freese, J., and Druckman, J. N. (2016). The demographic and political composition of mechanical turk samples. *Sage Open* 6, 2158244016636433

New York Post (2017). Us military will have more combat robots than human soldiers by 2025 Https://nypost.com/2017/06/15/us-military-will-have-more-combat-robots-than-human-soldiers-by-2025/

Oh, S. L., Hagiwara, Y., Raghavendra, U., Yuvaraj, R., Arunkumar, N., Murugappan, M., et al. (2018). A deep learning approach for parkinson's disease diagnosis from eeg signals. *Neural Computing and Applications* , 1–7

Palan, S. and Schitter, C. (2018). Prolific. ac—a subject pool for online experiments. *Journal of Behavioral and Experimental Finance* 17, 22–27

Solis, G. D. (1989). *Marines and Military Law in Vietnam: Trial by Fire* (History and Museums Division, Headquarters, US Marine Corps)

Sparrow, R. (2007). Killer robots. *Journal of applied philosophy* 24, 62–77

Tomasik, B. (2013). International cooperation vs. ai arms race. *Foundational Research Institute* 5

Tucker, S. C. (2011). *The Encyclopedia of the Vietnam War: A Political, Social, and Military History, [4 Volumes]: A Political, Social, and Military History* (Abc-clio)

Walters, K., Christakis, D. A., and Wright, D. R. (2018). Are mechanical turk worker samples representative of health status and health behaviors in the us? *PloS one* 13, e0198835
